# Angiography-Derived FFR Versus IVUS to Guide PCI According to Angiographic Lesion Characteristics

**DOI:** 10.1161/CIRCINTERVENTIONS.126.016809

**Published:** 2026-06-09

**Authors:** Seokhun Yang, Xinyang Hu, Jinlong Zhang, Jin-Eun Song, Jun Jiang, Xiaoping Peng, Dongsheng Lu, Yibin Pan, Lijun Guo, Jilin Li, Wenming He, Hao Zhou, Jun Pu, Jinyu Huang, Fan Jiang, Qiang Liu, Daqing Song, Liang Lu, Zhenfeng Cheng, Bin Yang, Jianliang Ma, Peng Chen, Shiqiang Li, Zhaohui Meng, Lijiang Tang, Yongzhen Fan, Eun-Seok Shin, Shenxian Tu, Chang-Wook Nam, William F. Fearon, Jian’an Wang, Bon-Kwon Koo

**Affiliations:** 1 Seoul National University Hospital, Republic of Korea (S.Y., J.-E.S., B.-K.K.).; 2 The Second Affiliated Hospital of Zhejiang University School of Medicine, State Key Laboratory of Transvascular Implantation Devices, Heart Regeneration and Repair Key Laboratory of Zhejiang Province, Transvascular Implantation Devices Research Institute, Hangzhou, China (X.H., J.Z., J.J., J.W.).; 3 Department of Cardiology, the First Affiliated Hospital of Nanchang University, Jiangxi Hypertension Research Institute, China (X.P.).; 4 Department of Cardiology, Changxing People’s Hospital, Huzhou, China (D.L.).; 5 Department of Cardiology, Jinhua Central Hospital, China (Y.P.).; 6 Department of Cardiology, Peking University Third Hospital, Key Laboratory of Cardiovascular Molecular Biology and Regulatory Peptides, Ministry of Health, Beijing, China (L.G.).; 7 Department of Cardiology, the Second Affiliated Hospital of Shantou University Medical College, China (J.L.).; 8 Department of Cardiology, the First Affiliated Hospital of Ningbo University, China (W.H.).; 9 Department of Cardiology, the First Affiliated Hospital of Wenzhou Medical University, China (H.Z.).; 10 Department of Cardiology, Renji Hospital, School of Medicine, Shanghai Jiao Tong University, China (J.P.).; 11 Department of Cardiology, Hangzhou First People’s Hospital, China (J.H.).; 12 Department of Cardiology, the Affiliated Hospital of Hangzhou Normal University, China (F.J.).; 13 Department of Cardiology, the Fourth People’s Hospital of Jinan, China (Q.L.).; 14 Department of Cardiology, Jining No.1 People’s Hospital, China (D.S.).; 15 Department of Cardiology, Dongyang People’s Hospital, Jinhua, China (L.L.).; 16 Department of Cardiology, Huzhou Central Hospital, China (Z.C.).; 17 Department of Cardiology, Second Hospital of Shanxi Medical University, Taiyuan, China (B.Y.).; 18 Department of Cardiology, the Affiliated Hospital of Shandong University of Traditional Chinese Medicine, Jinan, China (J.M.).; 19 Department of Cardiology, the Second Affiliated Hospital and Yuying Children’s Hospital of Wenzhou Medical University, China (P.C.).; 20 Department of Cardiology, Zhejiang Greentown Cardiovascular Hospital, Hangzhou, China (S.L.).; 21 Department of Cardiology, First Affiliated Hospital of Kunming Medical University, China (Z.M.).; 22 Department of Cardiology, Zhejiang Hospital, Hangzhou, China (L.T.).; 23 Department of Cardiology, Zhongnan Hospital of Wuhan University, China (Y.F.).; 24 Department of Cardiology, Ulsan University Hospital, University of Ulsan College of Medicine, Republic of Korea (E.-S.S.).; 25 Biomedical Instrument Institute, School of Biomedical Engineering, Shanghai Jiao Tong University, China (S.T.).; 26 Department of Cardiology, Keimyung University Dongsan Medical Center, Daegu, Republic of Korea (C.-W.N.).; 27 Division of Cardiovascular Medicine and Stanford Cardiovascular Institute, Stanford University School of Medicine, Stanford, and the VA Palo Alto Health Care System, CA (W.F.F.).

**Keywords:** angiography, coronary artery disease, risk factors

## Abstract

**BACKGROUND:**

While angiography-derived fractional flow reserve (AngioFFR)- and intravascular ultrasound (IVUS)-guided percutaneous coronary intervention (PCI) yield similar outcomes, with AngioFFR associated with lower PCI rates, the relative clinical effectiveness of AngioFFR versus IVUS-guided PCI according to angiographic lesion characteristics remains unclear.

**METHODS:**

This post hoc analysis of the FLAVOUR II trial (Comparison of Angiography-Derived Fractional Flow Reserve- and Intravascular Ultrasound-Guided Intervention Strategy for Clinical Outcomes in Patients with Coronary Artery Disease) included patients with ≥50% stenosis randomized to AngioFFR- or IVUS-guided PCI. A composite lesion risk score was derived using a marginal Cox model-based linear predictor incorporating % diameter stenosis, lesion length, true bifurcation, ostial lesion, and heavy calcification. The primary end point was target vessel failure (TVF: cardiac death, target vessel myocardial infarction, and target vessel revascularization).

**RESULTS:**

Among 1726 patients, 884 (51.2%) underwent AngioFFR-guided PCI and 842 (48.8%) underwent IVUS-guided PCI. During a median 12-month follow-up, TVF occurred in 2.4% of AngioFFR-treated vessels and 1.9% of IVUS-treated vessels (*P*=0.50). TVF risk increased progressively with a higher composite lesion risk score (adjusted hazard ratio, 2.76 [95% CI, 1.35–5.65]). This association was more pronounced in the AngioFFR group (adjusted hazard ratio, 3.93 [95% CI, 1.79–8.63]) than in the IVUS group (adjusted hazard ratio, 1.62 [95% CI, 0.42–6.22]). Compared with IVUS guidance, AngioFFR-guided vessels with high-risk lesions (score >0.32) had a higher TVF rate (5.1% versus 1.9%; *P*=0.010), whereas outcomes were comparable in those with low-risk lesions (1.5% versus 1.9%; *P*=0.531). AngioFFR guidance was associated with lower target vessel PCI rates in low-risk lesions (60.1% versus 76.5%; *P*<0.001), whereas PCI rates were uniformly high and similar between groups in high-risk lesions (96.4% versus 97.4%; *P*=0.675).

**CONCLUSIONS:**

A higher composite lesion risk score was associated with increased PCI rates and TVF risk after AngioFFR- or IVUS-guided treatment. AngioFFR guidance was associated with lower PCI rates in low-risk lesions, whereas IVUS guidance may yield more favorable outcomes in high-risk lesions.

**REGISTRATION:**

URL: https://www.clinicaltrials.gov; Unique identifier: NCT04397211.

**What is Known**

- In a head-to-head comparison between angiography-derived fractional flow reserve- and intravascular ultrasound-guided percutaneous coronary intervention (PCI), overall 1-year clinical outcomes were comparable, with a lower PCI rate in the angiography-derived fractional flow reserve-guided strategy.
- The relative clinical benefit according to angiographic lesion characteristics has not been fully understood.

**What the Study Adds**

- In lesions with lower angiographic risk, angiography-derived fractional flow reserve guidance reduced PCI rates without compromising 1-year clinical outcomes compared with intravascular ultrasound guidance.
- In lesions with higher angiographic risk, intravascular ultrasound guidance was associated with more favorable clinical outcomes despite similarly high PCI rates.
- These findings support a tailored selection of physiology- versus imaging-guided PCI according to angiographic lesion characteristics in daily practice.

In patients with coronary artery disease, coronary physiology–guided percutaneous coronary intervention (PCI)^1^ or intravascular imaging (IVI)–guided PCI^2,3^ is associated with a lower risk of future adverse clinical events compared with angiography-guided PCI alone, forming a cornerstone of contemporary state-of-the-art PCI.^4^ Recently, wireless physiological assessment using angiography-derived fractional flow reserve (AngioFFR) has demonstrated effectiveness in identifying ischemia-causing lesions and prognostic benefit in reducing major adverse cardiovascular events.^5,6^ While physiological assessment helps avoid unnecessary procedures by identifying functionally significant stenoses and IVI improves post-PCI outcomes through stent optimization, AngioFFR further allows post-PCI physiological evaluation to refine procedural results,^7^ whereas IVI provides detailed preprocedural assessment of lumen dimensions and plaque morphology.^8^ Building on these complementary capabilities, the recent FLAVOUR II trial (Comparison of Angiography-Derived Fractional Flow Reserve- and Intravascular Ultrasound-Guided Intervention Strategy for Clinical Outcomes in Patients with Coronary Artery Disease) showed that an AngioFFR-guided strategy applied from PCI decision-making through procedural optimization was noninferior to an intravascular ultrasound (IVUS)-guided strategy for clinical outcomes while being associated with a lower rate of PCI.^9^ Nonetheless, because the current guidelines recommend physiology-based decision-making for intermediate stenosis and IVI guidance for stent optimization in complex PCI,^10^ reflecting their use in different clinical contexts, clarifying how each strategy performs across varying lesion characteristics and complexity through a head-to-head comparison is essential for a more tailored approach. Therefore, we conducted a post hoc analysis of the FLAVOUR II trial to evaluate whether the relative clinical effectiveness of AngioFFR- versus IVUS-guided PCI differs according to angiographic lesion characteristics.

## Methods

### Data Sharing

Individual patient data will not be available to others. Data are available to the collaborators and will be shared upon reasonable request to the corresponding author.

### Study Design and Participants

FLAVOUR II was an investigator-initiated, open-label, multicenter, randomized, noninferiority trial designed to compare AngioFFR-guided and IVUS-guided PCI strategies with respect to 12-month clinical outcomes. Detailed aspects of the trial design have been published previously.^9,11^ Eligible patients were 18 years of age or older with a de novo coronary stenosis of ≥50% in a vessel with a visually estimated diameter of at least 2.5 mm on coronary angiography. Key exclusion criteria included the presence of a noncardiac comorbidity associated with a life expectancy of <1 year, target lesions located in the left main coronary artery or within a coronary artery bypass graft, and lesions in a totally occluded vessel. Among 1839 patients randomized between May 29, 2020, and September 20, 2023, 1726 who had full information on lesion characteristics were included in the current study (Figure S1). The trial was conducted in accordance with the International Council for Harmonisation Good Clinical Practice guidelines and the principles outlined in the Declaration of Helsinki. The protocol received approval from the institutional review board at each participating site, and all patients provided written informed consent. Oversight of the study was provided by an independent data and safety monitoring board, while all clinical events were adjudicated in a blinded fashion by an independent clinical events committee. The steering committee and all authors affirmed the accuracy and completeness of the data and confirmed that the conduct of the trial adhered to the approved protocol.

### Procedures

In the AngioFFR group, revascularization was indicated for vessels with AngioFFR ≤0.80. AngioFFR was assessed using either the quantitative flow ratio (Shanghai Pulse Medical Technology, Shanghai, China) or the Murray law–based quantitative flow ratio (Shanghai Pulse Medical Technology, Shanghai, China).^12–14^ In the IVUS group, revascularization was performed for lesions with a minimal lumen area ≤3.0 mm^2^ or a minimal lumen area >3.0 mm^2^ and ≤4.0 mm^2^ accompanied by a plaque burden >70%. Independent core laboratories analyzed all raw data: AngioFFR analyses were conducted at the CardHemo Core Laboratory, Med-X Research Institute, Shanghai Jiao Tong University, and IVUS analyses at Ulsan University Hospital. In both groups, data for core laboratory review were collected after the full procedure and PCI had been completed. Quantitative coronary angiography was conducted at the angiographic core laboratory of Seoul National University Hospital.

### Outcomes

Clinical outcomes were analyzed on a per-vessel basis. The primary outcome was target vessel failure (TVF), a composite end point of cardiac death, target vessel myocardial infarction, or target vessel revascularization at 12 months. Key secondary outcomes included each component of the primary end point and the cumulative target vessel PCI rate at the index procedure and throughout follow-up.

### Composite Lesion Risk Score and HRLC

The composite lesion risk score was quantified as a single continuous variable using a marginal Cox model-derived linear predictor using angiographic lesion characteristics, including quantitative coronary angiography-based % diameter stenosis and lesion length, as well as complex lesion features such as ostial lesion involving a major epicardial coronary artery, heavy calcification, defined as readily identifiable radiopacities within the vessel wall on angiography, or true bifurcation according to the Medina classification with a side-branch diameter ≥2.5 mm.^15–18^ A high composite lesion risk score was defined using a Youden index derived from receiver operating characteristic analysis. To improve interpretability, high-risk lesion characteristics (HRLC) were additionally analyzed using binary definitions (% diameter stenosis ≥70%, diffuse long coronary lesion [lesion length ≥38 mm], ostial lesion, heavy calcification, and true bifurcation) as a sensitivity analysis. All angiographic assessments were adjudicated by the angiographic core laboratory of Seoul National University Hospital.

### Statistical Analysis

The rationale and methodology for estimating the sample size for the FLAVOUR II trial have been detailed in earlier publications.^9,11^ Continuous variables were assessed for normality using the Shapiro-Wilk test and summarized as means with standard deviations or medians with interquartile ranges, as appropriate, while categorical variables were presented as counts with percentages. Comparisons between groups were performed using the Student *t* test or Wilcoxon rank-sum test for continuous variables and the χ^2^ test or Fisher exact test for categorical variables, as appropriate. Trends in proportions were evaluated using the Cochran-Armitage trend test. Vessel-specific clinical outcomes were analyzed using marginal Cox proportional hazards regression to account for within-patient correlation, yielding hazard ratios (HRs) and 95% CIs. The association between composite lesion risk score and the cumulative rate of target vessel PCI was evaluated using a generalized estimating equation model that adjusted for within-patient vessel-level correlation. A high composite lesion risk score was defined using the Youden index derived from receiver operating characteristic analysis. All analyses were conducted in the intention-to-treat population. A 2-sided *P*≤0.05 was considered statistically significant. To account for multiple comparisons, *P* values were adjusted using the Benjamini-Hochberg method to control the false discovery rate. All statistical analyses were performed using R software (version 4.3.1, R Foundation for Statistical Computing). The trial is registered at ClinicalTrials.gov (NCT04397211).

### Role of Funding Source

The funder of the study was not involved in the design of the study, collection or analysis of data, interpretation of results, or preparation of the article.

## Results

### Baseline Patient and Lesion Characteristics

Baseline clinical characteristics are summarized in Table 1. Among the 1726 patients, the mean age was 65.0±10.3 years, and 67.8% were male. A total of 884 (51.2%) patients underwent AngioFFR-guided PCI, and 842 (48.8%) patients underwent IVUS-guided PCI. The mean composite lesion risk score was similar between groups (0.12±0.39 versus 0.11±0.37 for AngioFFR and IVUS, respectively; *P*=0.772), and the prevalence of HRLC was also comparable. In the AngioFFR group, the frequencies of % diameter stenosis ≥70%, diffuse long coronary lesions, ostial lesion, heavy calcification, and true bifurcation lesion were 27.5%, 13.9%, 3.3%, 14.8%, and 7.6%, respectively, which were similar to those in the IVUS group (25.1%, 13.5%, 3.0%, 14.0%, and 6.8%).

**Table 1.**
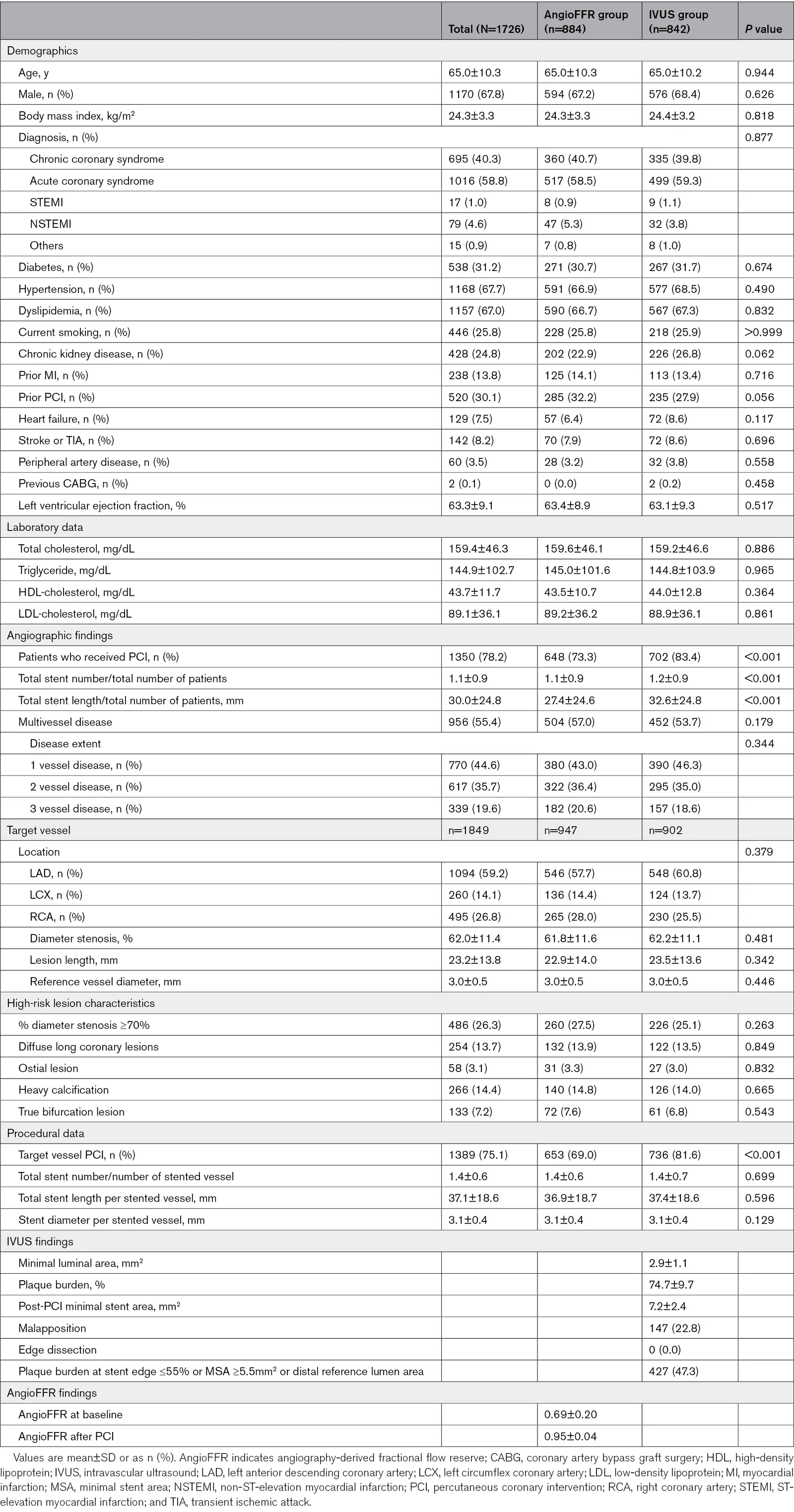
Baseline Characteristics

### TVF According to Lesion Risk

**Figure 1.**
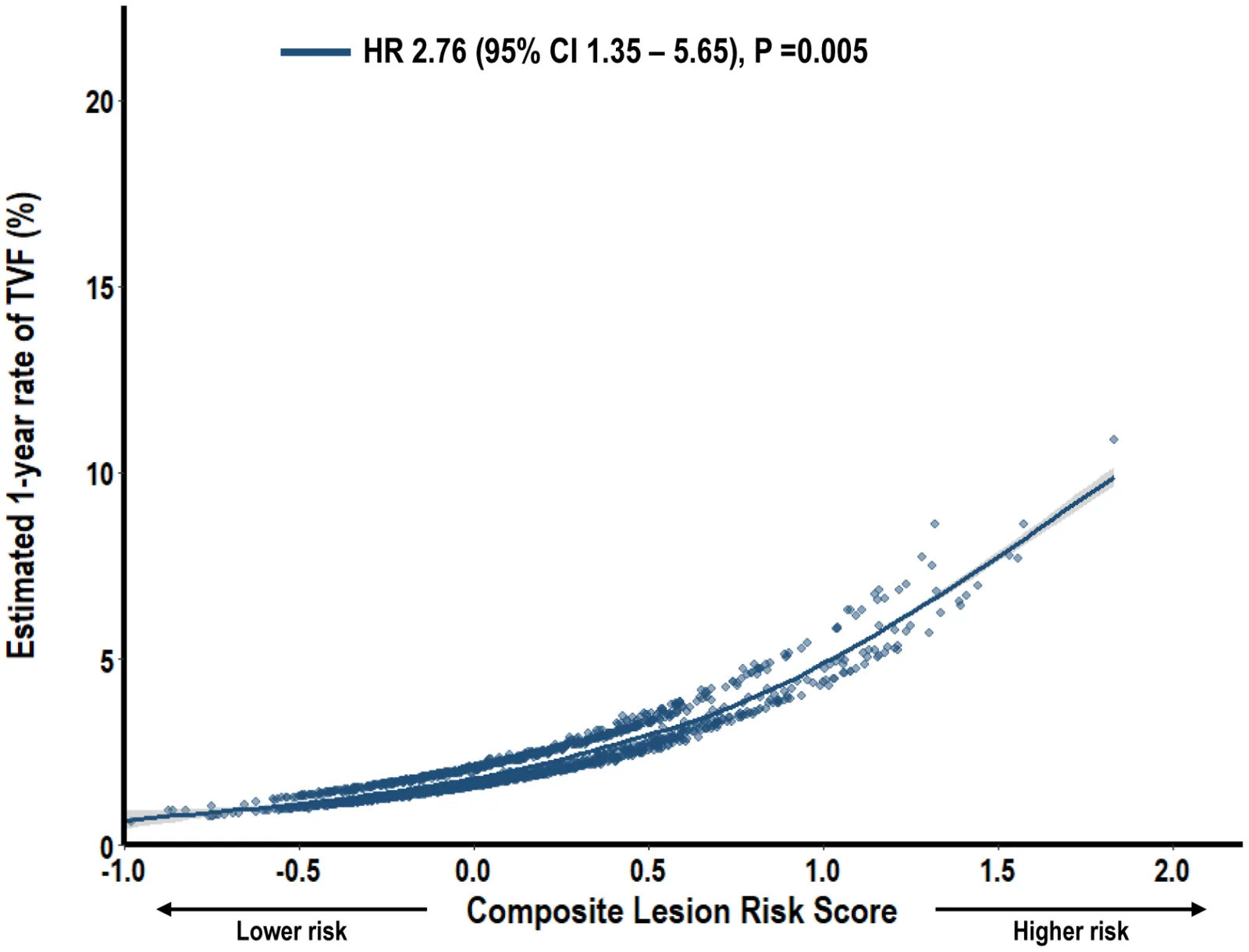
**Estimated 1-year risk of target vessel failure (TVF) according to lesion risk in total vessels.** Estimated 1-year risk of TVF is shown across the continuum of composite lesion risk score, represented by a marginal Cox model-derived linear predictor incorporating % diameter stenosis, lesion length, ostial lesion, heavy calcification, and true bifurcation. Hazard ratio (HR) was adjusted by left anterior descending artery and use of Murray law–based quantitative flow ratio.

During a median follow-up of 12 months, TVF occurred in 39 (2.2%) vessels, including 17 (1.9%) vessels in the IVUS group and 22 (2.4%) vessels in the AngioFFR group (HR, 1.25 [95% CI, 0.66–2.38]; *P*=0.495). Increasing composite lesion risk score was associated with a significantly higher estimated 1-year TVF rate (HR, 2.72 [95% CI, 1.33–5.57]; *P*=0.006), and this association remained significant after multivariable adjustment (adjusted HR, 2.76 [95% CI, 1.35–5.65], *P*=0.005; Figure 1). A similar trend was observed when lesion risk was assessed using binary criteria. As the number of HRLC increased, the cumulative event of TVF increased (1.7%, 1.9%, and 4.6% for vessels with 0, 1, and ≥2 HRLC, respectively; *P*-for-trend=0.014). In multivariable analysis, those with ≥2 HRLC demonstrated a significantly higher risk of TVF (adjusted HR, 2.77 [95% CI, 1.30–5.89], *P*=0.016; Table 2). Among the components of TVF, this association was predominantly driven by target vessel revascularization, which increased in parallel with the number of HRLC (1.2%, 1.4%, and 3.9% for 0, 1, and ≥2 HRLC, respectively; *P*-for-trend=0.009; Table S1).

**Table 2.**
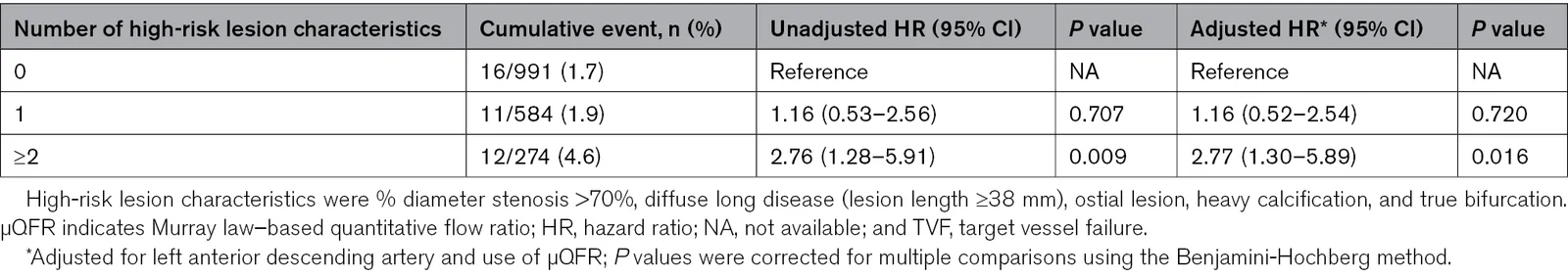
Association of High-Risk Lesion Characteristics With TVF

### Differential TVF Risk According to Lesion Risk in AngioFFR Versus IVUS-Guided Treatment Groups

**Figure 2.**
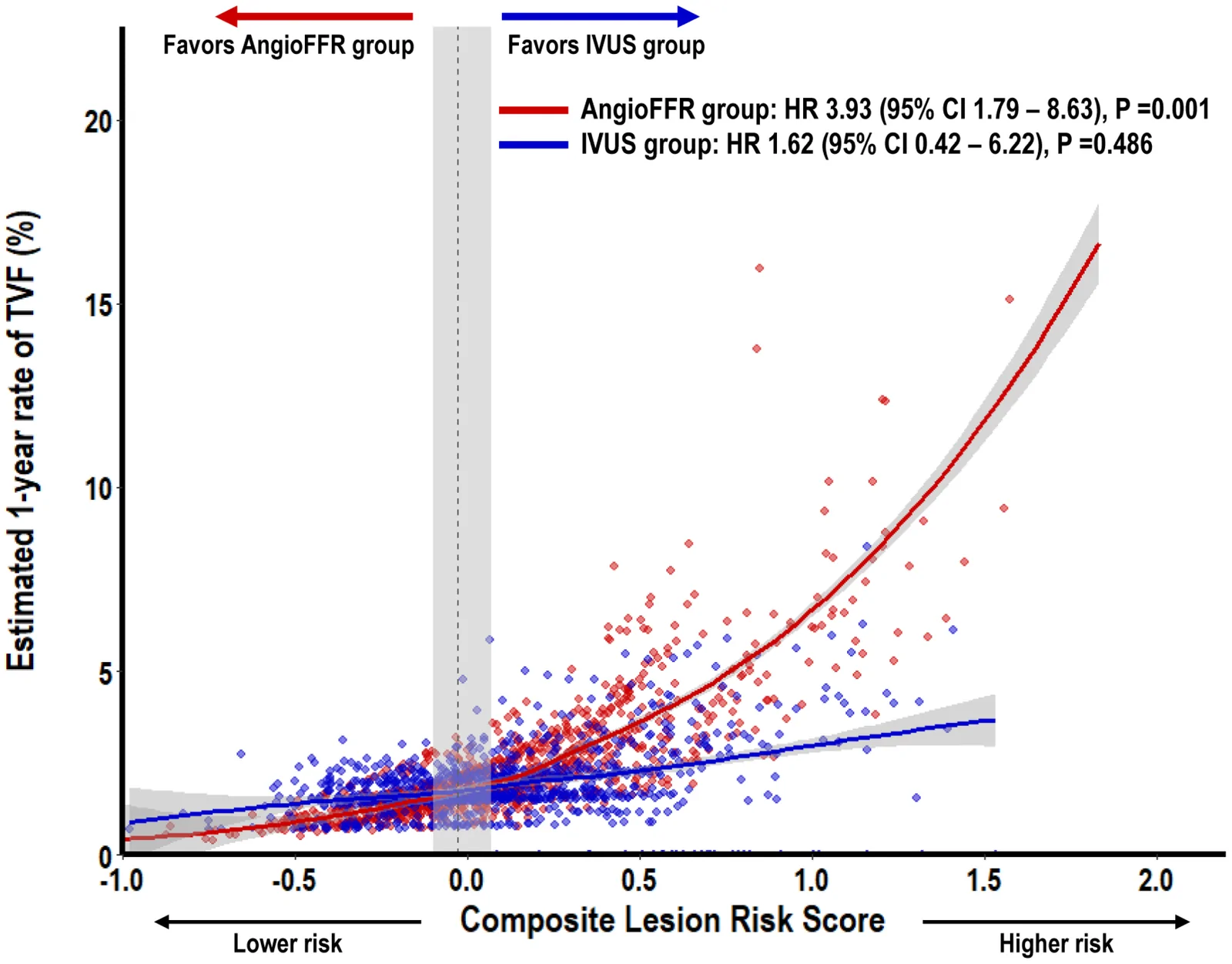
**Estimated 1-year risk of target vessel failure (TVF) according to lesion risk in angiography-derived fractional flow reserve (AngioFFR)- vs intravascular ultrasound (IVUS)-guided treatment.** Estimated 1-year risks of TVF in the AngioFFR and IVUS groups are depicted across the continuum of composite lesion risk score, represented by a marginal Cox model-derived linear predictor incorporating % diameter stenosis, lesion length, ostial lesion, heavy calcification, and true bifurcation. The vertical dashed line denotes the intersection point between the 2 curves, representing the score threshold at which the relative benefit shifts, while the shaded area indicates the 95% CI for this intersection point. Hazard ratio (HR) was adjusted by left anterior descending artery and use of Murray law–based quantitative flow ratio.

**Figure 3.**
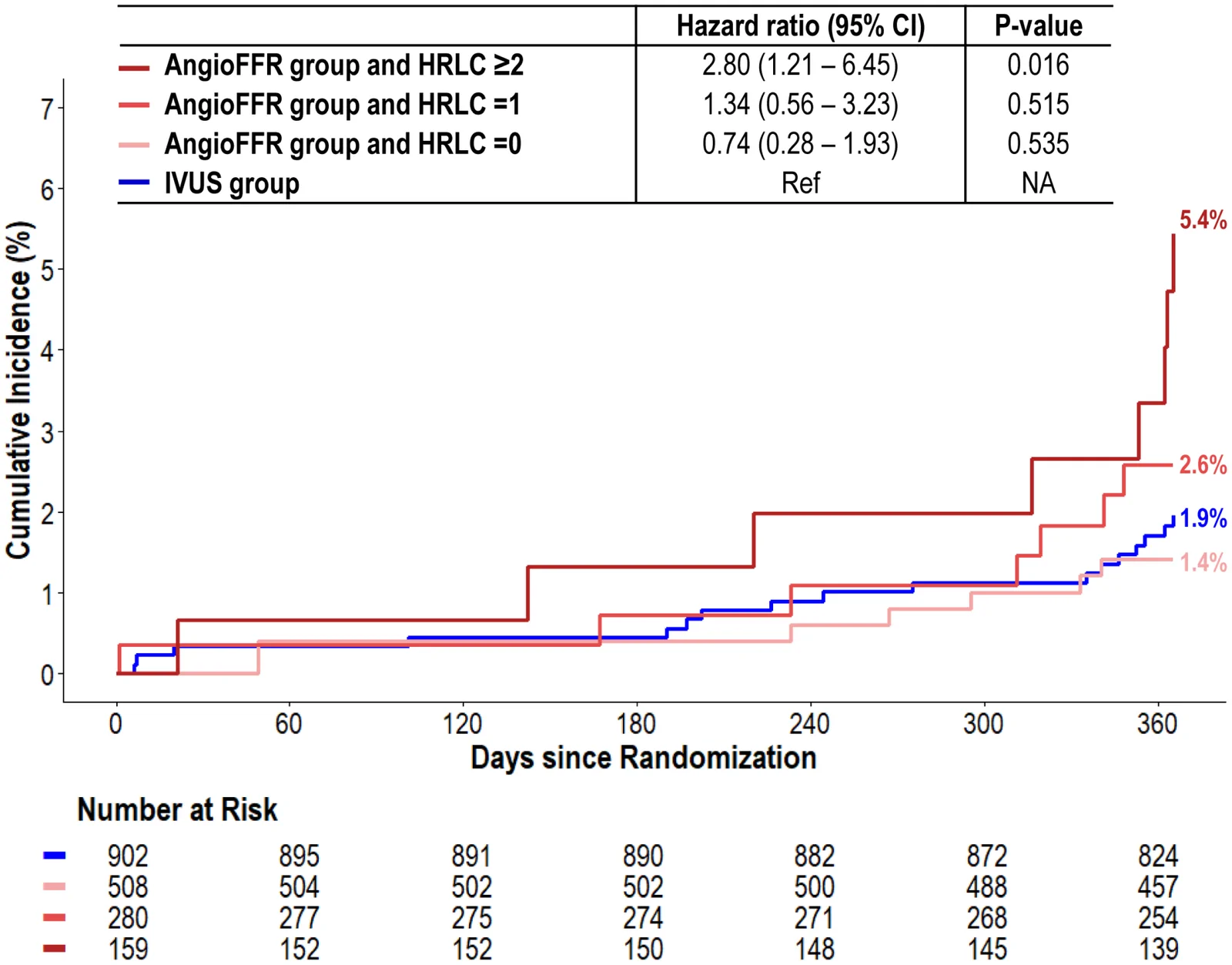
**Cumulative event rate of 1-year target vessel failure (TVF) according to high-risk lesion characteristics (HRLC) in the angiography-derived fractional flow reserve (AngioFFR) group vs the intravascular ultrasound (IVUS) group.** Cumulative event rate of 1-year TVF is shown for the AngioFFR group stratified by HRLC (0, 1, or ≥2) compared with the IVUS group. HRLC included % diameter stenosis >70%, diffuse long disease (lesion length ≥38 mm), ostial lesion, heavy calcification, and true bifurcation.

When the relationship between composite lesion risk score and the risk of TVF was evaluated separately in vessels treated with AngioFFR- versus IVUS-guided strategies, TVF risk increased exponentially with increasing composite lesion risk score in the AngioFFR group (adjusted HR, 3.93 [95% CI, 1.79–8.63]; *P*=0.001), whereas this association was attenuated and not statistically significant in the IVUS group (adjusted HR, 1.62 [95% CI, 0.42–6.22], *P*=0.486; *P*-for-interaction=0.312; Figure 2). The 2 fitted curves intersected at a composite lesion risk score of −0.03 (−0.10 to 0.07), and vessels with a composite lesion risk score >0.07 were classified as the IVUS-favoring subgroup, whereas vessels with a score ≤−0.10 were categorized as the AngioFFR-favoring subgroup. Compared with the AngioFFR-favoring group, the IVUS-favoring group demonstrated significantly higher % diameter stenosis (69.9±8.1% versus 50.1±7.6%; *P*<0.001), as well as higher proportions of heavy calcification (25.9% versus 1.2%; *P*<0.001), true bifurcation (14.6% versus 0.0%; *P*<0.001), and ostial lesions (6.4% versus 0.0%, *P*<0.001; Table S2). Bootstrap resampling (2000 iterations) demonstrated that the association between the composite lesion risk score and TVF was consistently observed in the AngioFFR group, with HR estimates predominantly above 1, whereas the corresponding estimates in the IVUS group more frequently included the null value, indicating a weaker association (Figure S2). Bootstrap internal validation additionally demonstrated an optimism-corrected C index of 0.61 and a calibration slope of 1.13. Consistent findings were observed using the number of HRLC (Figure S3). Compared with the IVUS group, AngioFFR-guided vessels with high composite lesion risk scores had a higher risk of TVF (HR, 2.64 [95% CI, 1.26–5.51]; *P*=0.010), whereas those with low-risk scores showed comparable outcomes (HR, 0.77 [95% CI, 0.34–1.76], *P*=0.531; Figure S4). When vessels were stratified according to the number of HRLC, vessels treated with the AngioFFR guidance with 0 or 1 HRLC showed no significant difference in the cumulative event of TVF compared with the IVUS-guided group, whereas those with ≥2 HRLC showed a significantly higher risk of TVF than IVUS-guided vessels (adjusted HR, 2.82 [95% CI, 1.22–6.50], *P*=0.045; Figure 3; Table 3). Within the IVUS group, stent malapposition, edge dissection, and optimization were comparable according to ≥2 HRLC, suggesting consistent procedural optimization regardless of lesion risk (Figure S5), and vessels with TVF in the AngioFFR group were more frequently associated with HRLC (Figure S6).

**Table 3.**
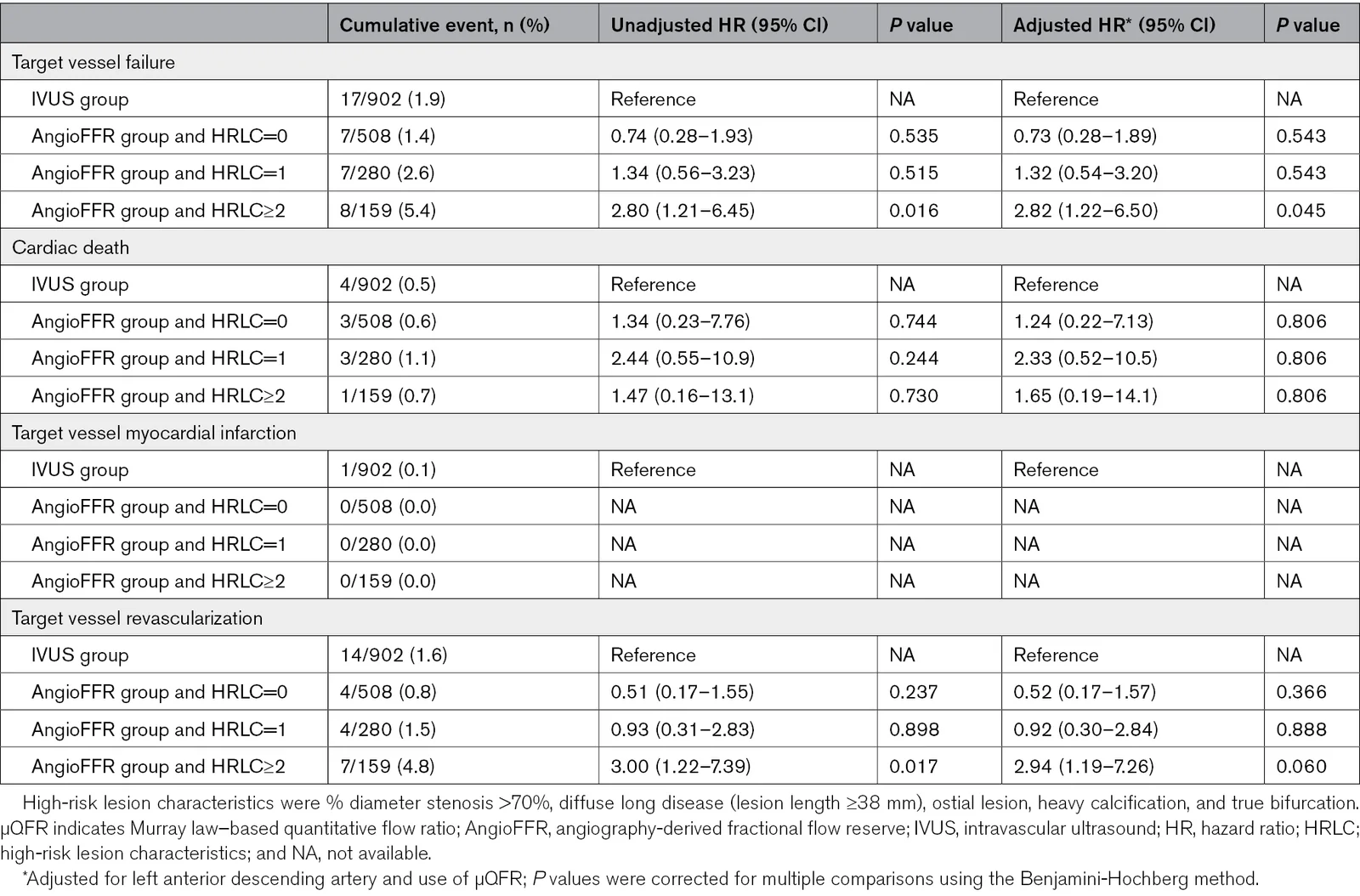
Clinical Outcomes After AngioFFR and IVUS-Guided Treatment According to High-Risk Lesion Characteristics

### Cumulative Rate of Target Vessel PCI According to Lesion Risk

**Figure 4.**
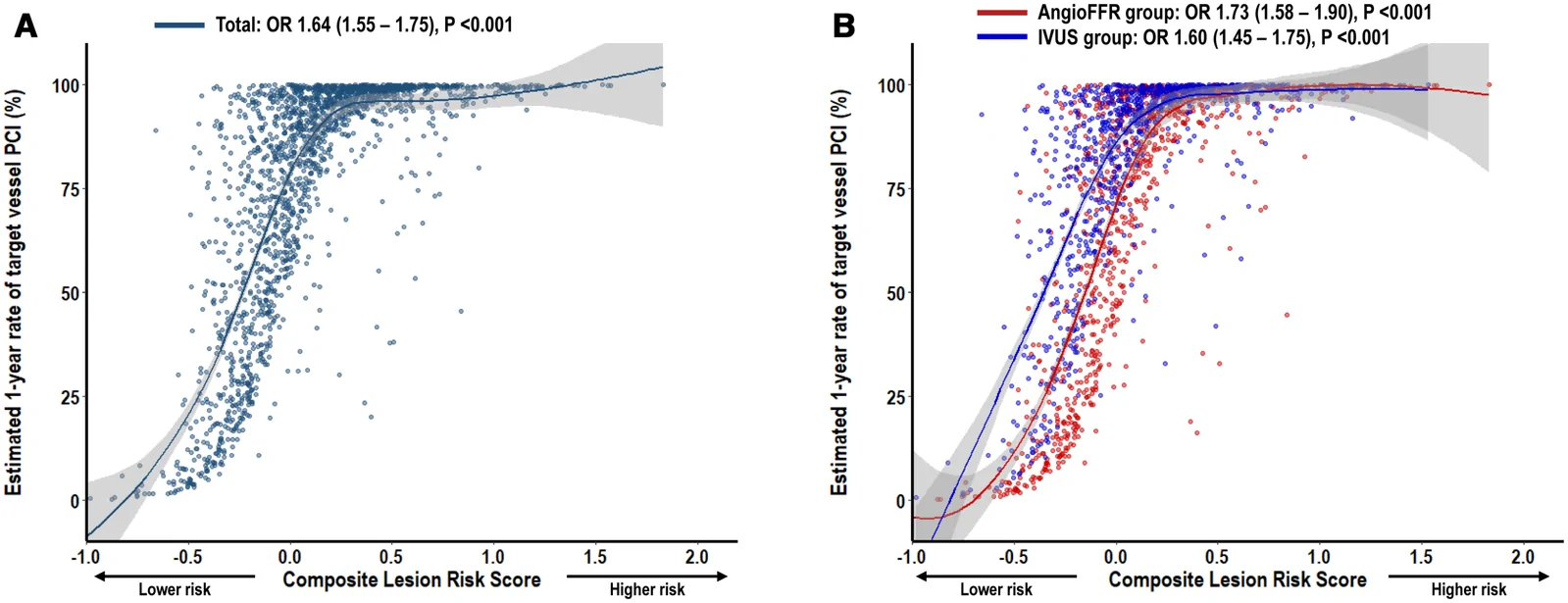
**Cumulative 1-year target vessel percutaneous coronary intervention (PCI) rate according to lesion risk.** Estimated cumulative 1-year rates of target vessel PCI, including PCI performed at the index procedure and during follow-up, are shown (**A**) for all vessels and (**B**) separately for the angiography-derived fractional flow reserve (AngioFFR) and intravascular ultrasound (IVUS) groups, according to composite lesion risk score quantified using a marginal Cox model-derived linear predictor incorporating % diameter stenosis, lesion length, ostial lesion, heavy calcification, and true bifurcation. Odds ratios (95% CI) per 10-unit increase in composite lesion risk score were estimated using a generalized estimating equation model. OR indicates odds ratio.

During the index procedure and 1-year follow-up, 1398 (75.6%) vessels underwent target vessel PCI, with significantly lower rates in the AngioFFR group compared with the IVUS group (69.6% versus 81.9%; *P*<0.001). Assessment across the spectrum of composite lesion risk score showed a significant increase in the cumulative rate of target vessel PCI with higher risk (Figure 4A), although the pattern varied by treatment strategy (Figure 4B). Vessels treated with the AngioFFR guidance showed a lower cumulative PCI rate at lower levels of lesion risk; however, this difference diminished with increasing lesion risk, and the 2 strategies demonstrated similar PCI rates at higher risk (Figure 4B). This observation was consistent across the number of HRLC. Among vessels with HRLC of 0, the AngioFFR group had a significantly lower 1-year target vessel PCI rate compared with the IVUS group (48.6% versus 70.6%; *P*<0.001). In contrast, among vessels with HRLC of 1 or ≥2, 1-year target vessel PCI rate were uniformly high and did not differ between the 2 groups (91.4% versus 93.1%; *P*=0.550 for HRLC of 1 and 98.1% versus 100.0%; *P*=0.372 for HRLC ≥2; Table 4).

**Table 4.**
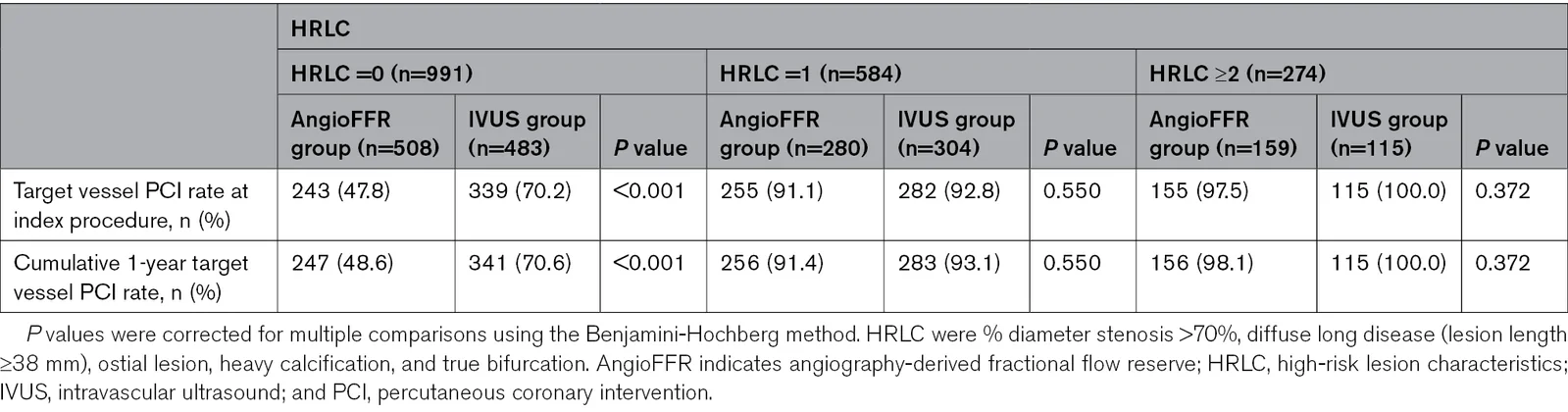
Cumulative Target Vessel PCI Rate According to High-Risk Lesion Characteristics

## Discussion

This post hoc analysis of the FLAVOUR II trial evaluated patients with coronary artery disease randomized to AngioFFR- or IVUS-guided PCI, stratified by lesion characteristics and complexity. First, the risk of TVF progressively increased with higher composite lesion risk scores. Second, when stratified by treatment strategy, increasing composite lesion risk score was associated with a significant increase in TVF risk in the AngioFFR group, whereas this pattern was attenuated in the IVUS group, resulting in a significantly higher risk of TVF among vessels treated with AngioFFR guidance with ≥2 HRLC compared with the IVUS group. Third, although AngioFFR guidance was associated with a lower overall rate of target vessel PCI, this PCI-sparing effect was mainly observed in lesions with lower composite lesion risk scores, whereas PCI rates became uniformly high and comparable to IVUS guidance as composite lesion risk score increased.

### Clinical Relevance of Lesion Characteristics in Patients Undergoing AngioFFR- Versus IVUS-Guided PCI

Physiological assessment is essential for identifying lesions that may benefit from revascularization, and AngioFFR has expanded its feasibility by providing wire-free and hyperemia-free measurements with acceptable diagnostic performance.^6^ In addition, AngioFFR enables pre- and post-PCI functional assessment of disease distribution, which can help refine post-PCI physiological results.^7^ IVI, on the other hand, facilitates accurate stent sizing and optimization by visualizing stent expansion, malapposition and edge dissection,^19^ and recent studies have further consolidated its efficacy in preprocedural lesion characterization for revascularization decisions.^8,20^ In the FLAVOUR II trial, these 2 strategies showed comparable clinical outcomes across both PCI decision-making and procedural optimization.^9^ Meanwhile, in the contemporary PCI era, baseline angiographic lesion profiles remain prognostic determinants of future coronary events. In the IRIS-FFR registry (Interventional Cardiology Research In-cooperation Society Fractional Flow Reserve), increasing % diameter stenosis was associated with a higher risk of clinical events even after accounting for fractional flow reserve values, with % diameter stenosis ≥70% conferring approximately a 2.5-fold higher risk.^18^ Following drug-eluting stent implantation, long stenting (≥40 mm) has been associated with nearly a 2-fold higher risk of target lesion failure.^21^ Moreover, in a subanalysis of the RENOVATE-COMPLEX PCI trial (Randomized Controlled Trial of Intravascular Imaging Guidance vs Angiography-Guidance on Clinical Outcomes After Complex Percutaneous Coronary Intervention), which randomized patients undergoing complex PCI to IVI- versus angiography-guided PCI, the risk of TVF was about 1.5× higher in patients with at least 3 complex lesion features compared with those with fewer features.^22^ These observations highlight the continued relevance of composite lesion risk as a prognostic marker even in the modern era of PCI. In this context, although AngioFFR- and IVUS-guided PCI resulted in similar clinical outcomes in the FLAVOUR II trial, understanding how these strategies perform across different lesion profiles is essential for refining treatment selection and informing a more individualized approach to PCI guidance. Therefore, we performed this post hoc analysis of the FLAVOUR II trial to assess the clinical benefit of the 2 strategies according to lesion characteristics.

### Differential Outcome Trends of AngioFFR- and IVUS-Guided PCI According to Lesion Risk

Previous studies have shown that AngioFFR can reliably assess functional significance in subsets including bifurcation, heavy calcification, and tandem disease,^23^ and that it may assist procedural optimization through post-PCI physiological assessment in high-risk indicated procedures including diffuse long lesions, true bifurcations and severe calcification.^7^ Despite this potential benefit of AngioFFR in complex lesions, no prior study had directly compared AngioFFR with IVUS, which is the guideline-recommended strategy for complex PCI.^10^ In this analysis, lesion risk was defined using angiographic lesion characteristics (% diameter stenosis, lesion length) and complex lesion features (true bifurcation, ostial location, heavy calcification), quantified both continuously using a linear predictor and categorically by HRLC criteria. Although higher composite lesion risk score was associated with higher rates of TVF, this outcome trend differed substantially by treatment strategy. The risk gradient was steeper in vessels treated with AngioFFR guidance, whereas the increase in risk was attenuated in vessels treated with IVUS guidance. Although the interaction between treatment strategy and lesion risk was not statistically significant, a significant association between lesion risk and TVF was observed only in the AngioFFR group, suggesting a potential differential pattern. Furthermore, AngioFFR-guided vessels without HRLC demonstrated the lowest rate of TVF at 1.4%, whereas those with ≥2 HRLC showed a significant higher TVF rate compared with the IVUS group (5.4% versus 1.9%; *P*=0.019). These findings align with prior evidence showing the benefit of IVI in complex lesion subsets, such as true bifurcation, ostial lesions, and heavily calcified lesions,^24–26^ and indicate that AngioFFR guidance is most preferable for vessels with lower lesion risk, whereas IVUS guidance provides greater clinical benefit in vessels with higher lesion risk, in terms of TVF, consistent with current clinical practice patterns. Notably, the FLAVOUR II trial design integrates both diagnostic thresholds and procedural optimization criteria, and thus the observed differences reflect a combined diagnostic and treatment strategy. In this context, it should be recognized that wire-based post-PCI physiological assessment may provide more accurate evaluation than angiography-derived physiology, particularly in complex lesion subsets such as bifurcations or heavily calcified lesions, where angiographic assessment may be limited.

### Benefit of AngioFFR-Guided PCI in Reduction of Procedural Burden Across Lesion Risk

The efficacy of physiological assessment primarily derives from its ability to reduce unnecessary PCI and procedural burden by identifying lesions that are functionally significant and most likely to benefit from revascularization. This benefit has been consistently demonstrated over anatomy-based strategies, as shown in the FAME trial (Fractional Flow Reserve Versus Angiography for Multivessel Evaluation) against angiography-guided PCI and in the FLAVOUR trial against IVUS-guided PCI.^1,20^ Similarly, the FLAVOUR II trial confirmed that AngioFFR guidance resulted in a lower overall PCI rate compared with IVUS guidance.^9^ However, it has remained unclear whether this PCI-sparing effect is preserved across the full spectrum of lesion risk. In the present analysis, AngioFFR guidance was associated with a lower cumulative PCI rate at lower levels of composite lesion risk score, consistent with the expected benefit of selectively treating functionally significant lesions. With increasing composite lesion risk score, however, this difference diminished and ultimately converged with IVUS-guided PCI rates. This finding aligns with the observation that increasing stenosis severity and morphological lesion complexity, such as eccentricity, bend, irregularity, calcification, bifurcation, and diffuseness, are associated with lower fractional flow reserve values and a higher likelihood of functional significance.^27,28^ As the level of angiographic lesion risk increases, revascularization becomes more likely regardless of whether decisions are guided by AngioFFR or IVUS. These observations suggest a shift in clinical priorities according to lesion risk. In low-risk lesions, the principal goal is accurate lesion selection, where AngioFFR confers the greatest benefit. In contrast, in vessels with higher lesion risk or complex anatomic features, procedural optimization may be more critical than lesion selection, favoring the use of IVUS. Practically, this implies that when treating high-risk lesions, clinicians should emphasize selecting the modality that best supports procedural optimization^3,15,16,25^ rather than relying solely on physiology-based lesion selection.

### Limitations

This study has several limitations. First, as it was a post hoc subgroup analysis of the FLAVOUR II trial that was not prespecified, the potential for multiplicity and chance findings cannot be excluded, and the findings should therefore be considered hypothesis-generating. Second, the follow-up period was restricted to 12 months, and longer-term data are needed to validate the current findings. Third, the overall event rate in the FLAVOUR II trial was lower than anticipated, which may have limited the statistical power of the current analysis, particularly for subgroup and component end point analyses that remain vulnerable to overfitting. In addition, competing-risk analyses were not performed because of the low number of cardiac deaths, and therefore death as a competing event for nonfatal outcomes could not be fully accounted for. Fourth, certain complex lesion features, including chronic total occlusions and left main disease, were not evaluated, as these lesions were excluded from the study population by design. Fifth, although internal validation using bootstrap resampling demonstrated consistent findings, external validation of the composite lesion risk score is required to confirm its generalizability. Furthermore, the cutoff value used to define high composite lesion risk was data derived using the Youden index and may therefore have limited clinical interpretability, requiring further validation. Sixth, the observed differences were largely driven by repeat revascularization rather than hard clinical end points and therefore do not support superiority in major clinical outcomes between treatment strategies. Seventh, because IVUS was not performed in the AngioFFR group, the underlying procedural mechanisms remain incompletely understood and warrant further investigation. Eighth, the generalizability of our findings may be limited, as the study population predominantly consisted of East Asian patients. In addition, exclusion of patients with insufficient angiographic imaging quality for core laboratory adjudication may have introduced potential selection bias. Finally, although both Murray law–based quantitative flow ratio and quantitative flow ratio were included in the analysis and adjustment for their use yielded consistent results, the findings may be influenced by the specific AngioFFR techniques used and require validation across other platforms.

### Conclusions

In this post hoc analysis of the FLAVOUR II trial, increasing composite lesion risk score was related to a higher TVF risk and target vessel PCI rate after both AngioFFR- and IVUS-guided PCI. In low-risk lesions, AngioFFR enabled lower PCI rates with outcomes comparable to IVUS guidance, whereas in high-risk lesions, IVUS guidance led to more favorable clinical outcomes despite similar PCI rates. These findings support using AngioFFR for lesion selection in low-risk lesions and IVUS for procedural optimization in high-risk lesions. However, the results should be considered hypothesis-generating, particularly given the nonsignificant interaction analysis, low event rate, and the predominance of repeat revascularization rather than hard clinical end points.

## Article Information

### Acknowledgments

The angiography-derived fractional flow reserve (AngioFFR) system was provided by Shanghai Pulse Medical Technology without charge. The authors thank all study patients accepting enrollment in the trial and contributors.

### Disclosures

Drs Wang and Hu report an institutional research grant from Boston Scientific Corporation and free licenses for the quantitative flow ratio (QFR) and Murray law–based quantitative flow ratio (µQFR) application from Pulse Medical during the trial. Dr Tu is the cofounder of, has received research grants from, and been a consultant (without payment) for Pulse Medical. Dr Nam reports an institutional research grant from Abbott. Dr Fearon reports institutional research grants from Abbott Vascular, CathWorks, and Medtronic; consulting fees from ShockWave; and stock or stock options from HeartFlow. Dr Koo reports institutional research grants from Abbott Vascular, Boston Scientific Corporation, and Philips. The other authors report no conflicts.

### Supplemental Material

Supplemental Methods

Tables S1–S2

Figures S1–S6

FLAVOUR II Study Group

## Supplementary Material
