# Supplementary material for: Angiography-Derived FFR Versus IVUS to Guide PCI According to Angiographic Lesion Characteristics
Source: Circ Cardiovasc Interv. 2026 Jun 9;19(9):e016809. doi: 10.1161/CIRCINTERVENTIONS.126.016809 (PMC13574350; doi:10.1161/CIRCINTERVENTIONS.126.016809)
Supplement: Supplementary file 1 [file hcv-19-e016809-s001.pdf]

SUPPLEMENTAL MATERIAL

Angiography-derived FFR versus IVUS to guide PCI according to angiographic lesion characteristics

On behalf of the FLAVOUR II investigators

Table of Contents

1. Supplemental Methods 3

2. Supplemental Figures and Figure Legends 7

Figure S1. Study flow 7

Figure S2. Cumulative incidence of PCI according to individual components of high-risk lesion characteristics 8

Figure S3. Cumulative incidence of TVF according to the number of high-risk lesion characteristics and treatment strategy 9

Figure S4. Cumulative event rate of 1-year TVF according to composite lesion risk score in the AngioFFR group compared with the IVUS group 10

Figure S5. Post-IVUS procedural findings according to ≥2 HRLC in the IVUS group 11

Figure S6. Distribution of lesion characteristics according to ≥2 HRLC among vessels with TVF in the AngioFFR group 12

3. Supplemental Tables 13

Table S1. Association of high-risk lesion characteristics with clinical outcomes 13

Table S2. Comparison of lesion characteristics between AngioFFR and IVUS-favoring subgroups 14

1. Supplemental Methods
Complete List of Investigators
1. The Second Affiliated Hospital of Zhejiang University School of Medicine, Hangzhou, China
   a. Jian'an Wang, MD
   b. Xinyang Hu, MD
   c. Jun Jiang, MD
   d. Xianbao Liu, MD
   e. Yong Sun, MD
   f. Changling Li, MD
   g. Bingchen Liu, MD
   h. Zhangping Yu, MD
   i. Wen'ai Chen, MD
   j. Liang Dong, MD
   k. Bin Nie, MD
   l. Yabin Liu, MD
   m. Feng Gao, MD
   n. Lingjun Zhu, MD
   o. Qiyuan Xu, MD
   p. Jing Li, MD
   q. Haibo Chen, MD
   r. Qiyue Gao, MD
   s. Qucheng Wei, MD
   t. Jinlong Zhang, MD
   u. Tonghui Zhao, MD

2. The First Affiliated Hospital of Nanchang University and Jiangxi Hypertension Research Institute, Nanchang, China
   a. Xiaoping Peng, MD
   b. Yu Jiang, MD
   c. Zhiliang Zhang, MD
   d. Wenyao Cai, MD
   e. Guorong Cai, MD
   f. Dingkun Wang, MD
   g. Wenbo Luo, MD

3. Changxing People's Hospital, Huzhou, China
   a. Dongsheng Lu, MD
   b. Ao Liu, MD

4. Jinhua Central Hospital, Jinhua, China
   a. Yibin Pan, MD
   b. Juncang Duan, MD

5. Peking University Third Hospital, Beijing, China
   a. Lijun Guo, MD
   b. Xinye Xu, MD

c. Liyun He, MD
d. Ming Cui, MD
e. Lingyun Zu, MD
f. Jiangli Han, MD
g. Yupeng Wang, MD
h. Lin Mi, MD
i. Juntao Duan, MD

6. The Second Affiliated Hospital of Shantou University Medical College, Shantou, China
   a. Jilin Li, MD
   b. Bin Xie, MD

7. The First Affiliated Hospital of Ningbo University, Ningbo, China
   a. Wenming He, MD
   b. Yanqing Xie, MD

8. The First Affiliated Hospital of Wenzhou Medical University, Wenzhou, China
   a. Hao Zhou, MD
   b. Changxi Chen, MD
   c. Zhan Gao, MD

9. Renji Hospital, Shanghai Jiao Tong University School of Medicine, Shanghai, China
   a. Jun Pu, MD
   b. Song Ding, MD

10. Hangzhou First People's Hospital, Hangzhou, China
   a. Jinyu Huang, MD
   b. Guoxin Tong, MD

11. The Affiliated Hospital of Hangzhou Normal University, Hangzhou, China
   a. Fan Jiang, MD
   b. Yukai Chen, MD

12. The Fourth People's Hospital of Jinan, Jinan, China
   a. Qiang Liu, MD
   b. Fangjie Jiao, MD

13. Jining No.1 People's Hospital, Jining, China
   a. Daqing Song, MD
   b. Ya Li, MD

14. Dongyang People's Hospital, Dongyang, China
   a. Liang Lu, MD
   b. Hongping Si, MD

15. Huzhou Central Hospital, Huzhou, China
   a. Zhenfeng Cheng, MD

b. Liying Shen, MD

16. Second Hospital of Shanxi Medical University, Taiyuan, China
   a. Bin Yang, MD
   b. Huili Cao, MD

17. The Affiliated Hospital of Shandong University of Traditional Chinese Medicine, Jinan, China
   a. Jianliang Ma, MD
   b. Zhen Wang, MD
   c. Yong Wang, MD

18. The Second Affiliated Hospital and Yuying Children's Hospital of Wenzhou Medical University, Wenzhou, China
   a. Peng Chen, MD

19. Zhejiang Greentown Cardiovascular Hospital, Hangzhou, China
   a. Shiqiang Li, MD
   b. Shen Wang, MD

20. First Affiliated Hospital of Kunming Medical University, Kunming, China
   a. Zhaohui Meng, MD
   b. Guihu Sun, MD
   c. Lihong Yang, MD
   d. Xiaona Yang, MD

21. Zhejiang Hospital, Hangzhou, China
   a. Lijiang Tang, MD
   b. Changqing Du, MD

22. Zhongnan Hospital of Wuhan University, Wuhan, China
   a. Yongzhen Fan, MD
   b. Zhibing Lu, MD
   c. Xiaorong Hu, MD

23. Seoul National University Hospital, Seoul, Republic of Korea
   a. Bon-Kwon Koo, MD
   b. Doyeon Hwang, MD
   c. Seokhun Yang, MD

24. Ulsan University Hospital, Ulsan, Republic of Korea
   a. Eun-Seok Shin, MD

25. Shanghai Jiao Tong University, Shanghai, China
   a. Shengxian Tu, PhD

26. Keimyung University Dongsan Medical Center, Daegu, Republic of Korea

a. Chang-Wook Nam, MD

27. Division of Cardiovascular Medicine and Stanford Cardiovascular Institute, Stanford University School of Medicine, Stanford, CA, USA
a. William F. Fearon, MD

2. Supplemental Figures and Figure Legends

Figure S1. Study flow

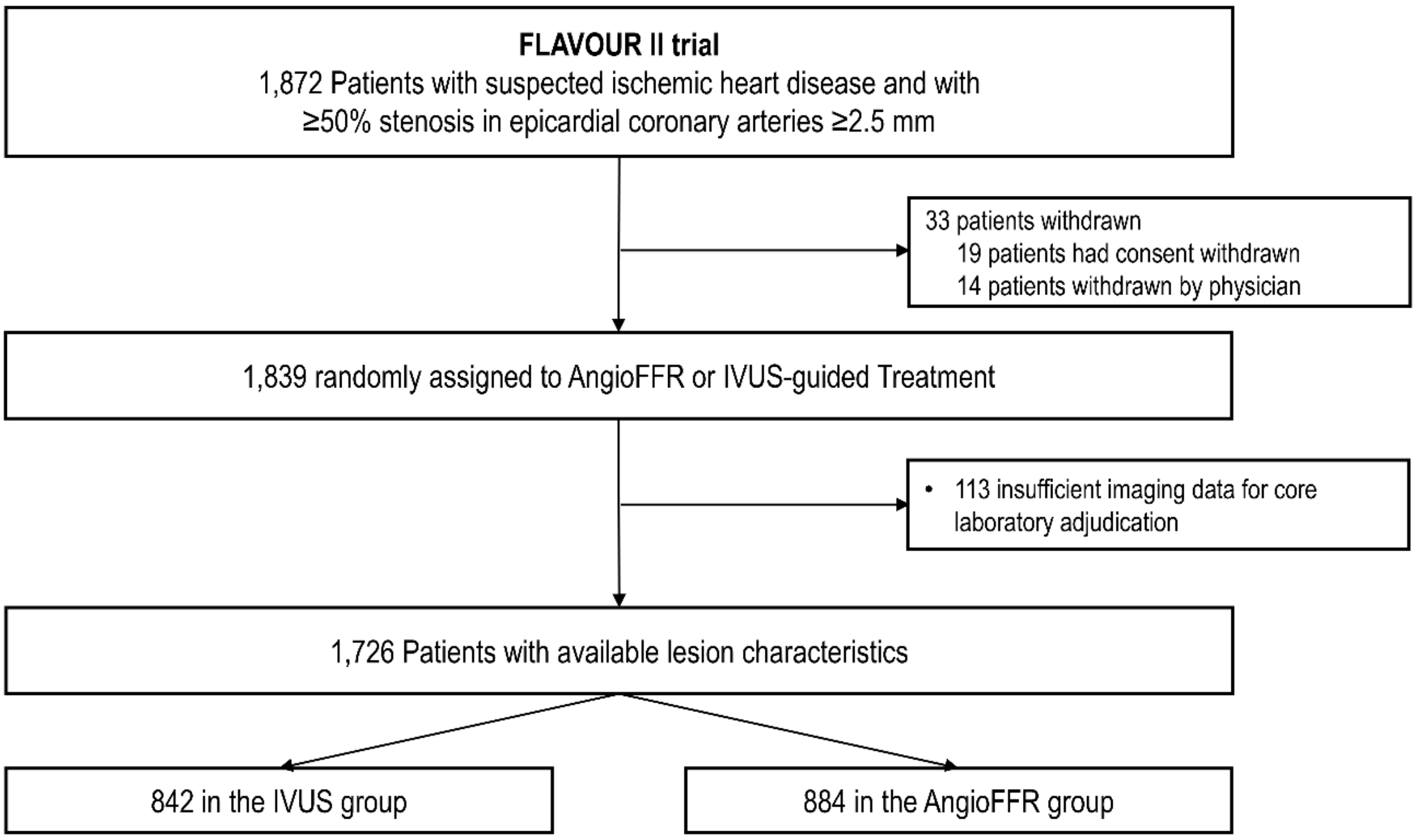

Flow diagram of patient enrollment, randomization, and inclusion in the present analysis of the FLAVOUR II trial is presented. Of 1,872 patients screened, 1,839 were randomized to AngioFFR- or IVUS-guided treatment. After excluding patients with insufficient imaging data for core laboratory adjudication, 1,726 patients with available lesion characteristics were included in the analysis.
Abbreviations: AngioFFR, angiography-derived fractional flow reserve; IVUS, intravascular ultrasound.

Figure S2. Bootstrap distribution of hazard ratios for the association between composite lesion risk score and TVF according to treatment strategy

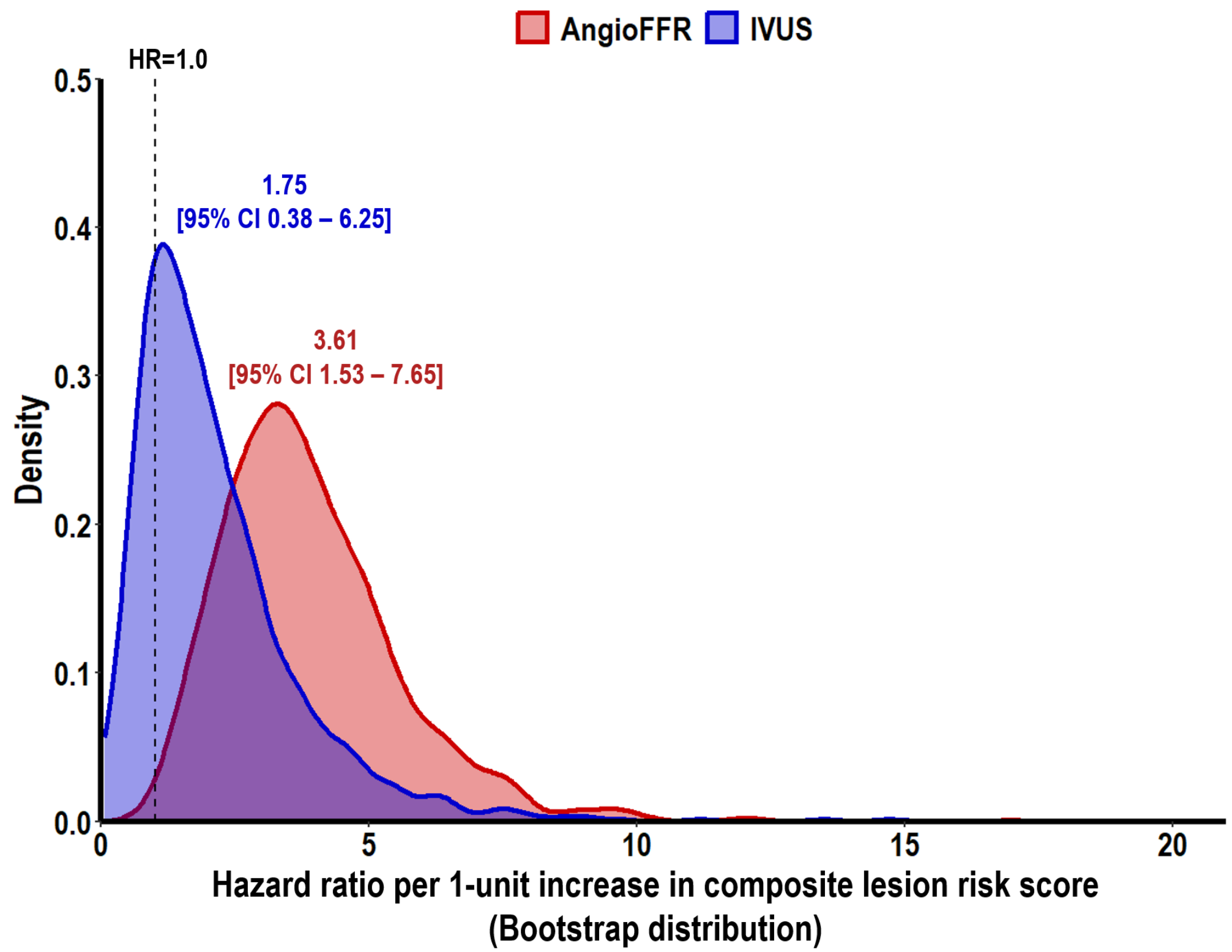

Distribution of hazard ratios for the association between composite lesion risk score and TVF derived from 2,000 bootstrap resampling iterations. The AngioFFR group demonstrates a consistent positive association, with hazard ratio estimates predominantly above 1, whereas the IVUS group shows greater variability with estimates frequently spanning the null value. Vertical dashed line indicates a hazard ratio of 1.0.

Abbreviations: AngioFFR, angiography-derived fractional flow reserve; CI, confidence interval; HR, hazard ratio; IVUS, intravascular ultrasound; TVF, target vessel failure.

Figure S3. Cumulative incidence of TVF according to the number of high-risk lesion characteristics and treatment strategy

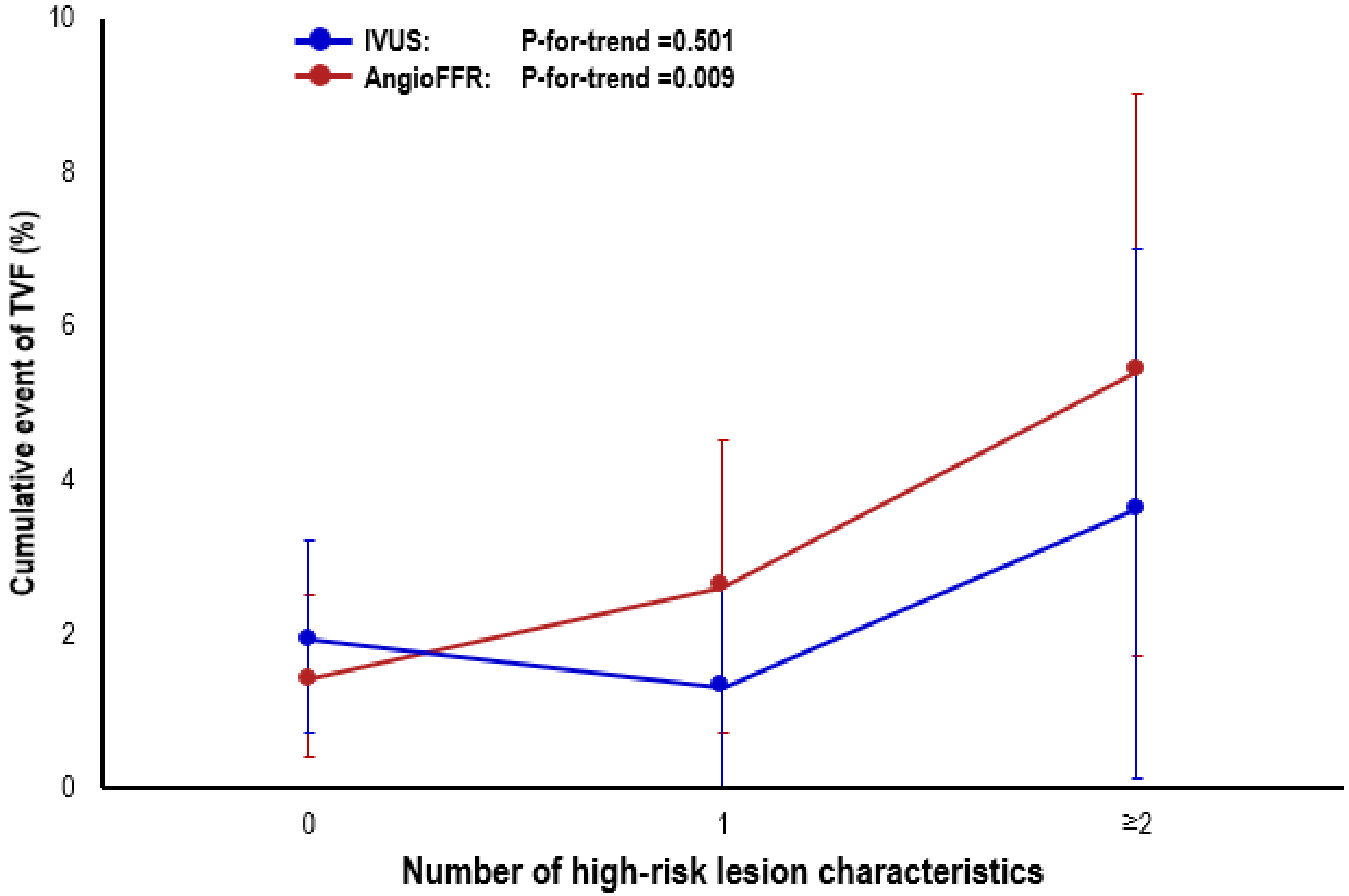

Cumulative incidence of TVF according to the number of high-risk lesion characteristics (0, 1, and ≥2) stratified by treatment strategy. Error bars indicate 95% confidence intervals. High-risk lesion characteristics were % diameter stenosis >70%, diffuse long disease (lesion length ≥38 mm), ostial lesion, heavy calcification, and true bifurcation. Abbreviations: AngioFFR, angiography-derived fractional flow reserve; IVUS, intravascular ultrasound; TVF, target vessel failure.

Figure S4. Cumulative event of 1-year TVF according to composite lesion risk score in the AngioFFR group compared with IVUS group

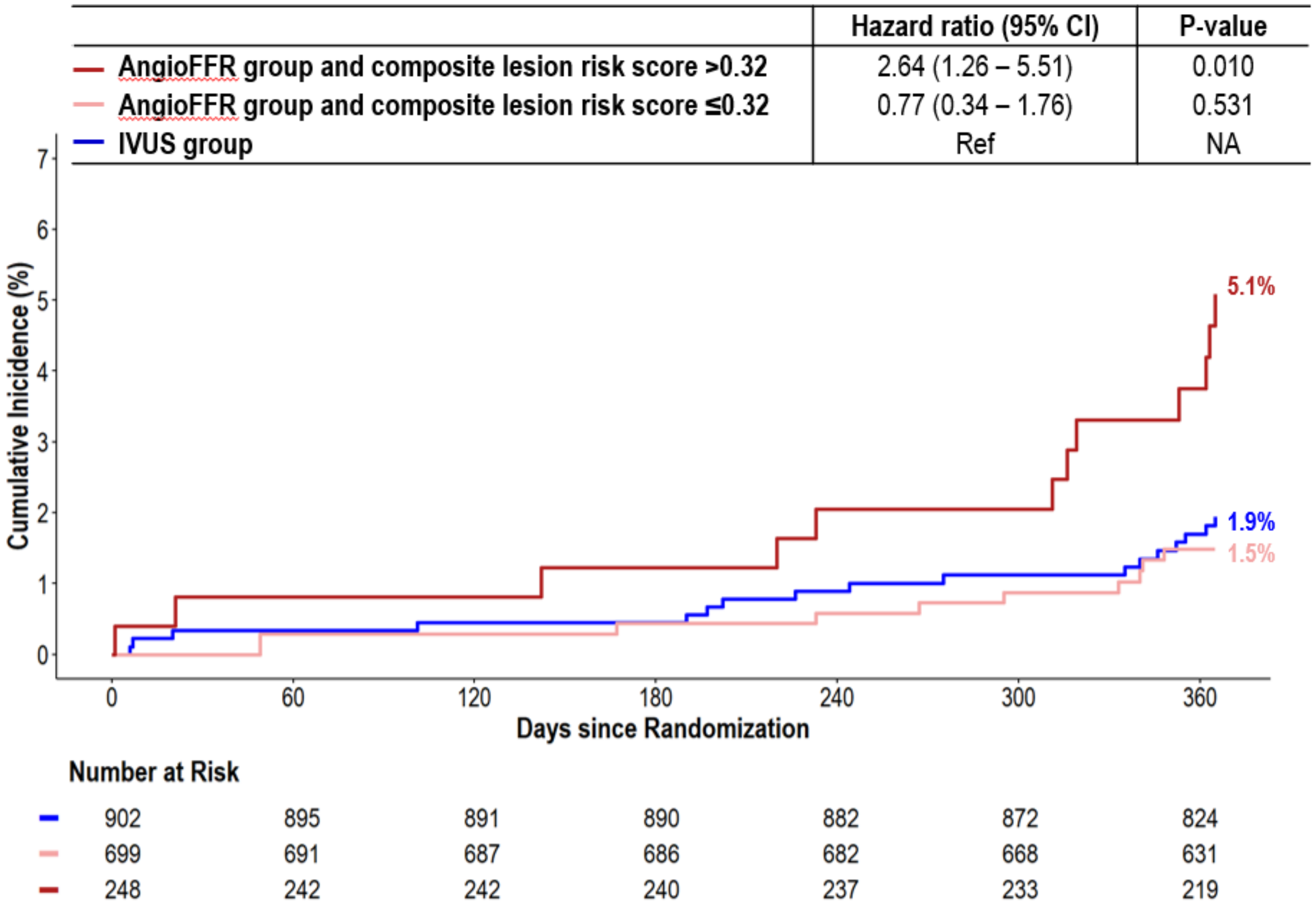

Cumulative event of 1-year TVF is presented for the AngioFFR group stratified by composite lesion risk score in comparison with the IVUS group. Composite lesion risk score was quantified using a marginal Cox model–derived linear predictor incorporating % diameter stenosis, lesion length, ostial lesion, heavy calcification, and true bifurcation.

Abbreviations: AngioFFR, angiography-derived fractional flow reserve; CI, confidence interval; IVUS, intravascular ultrasound.

Figure S5. Post-IVUS procedural findings according to ≥2 HRLC in the IVUS group

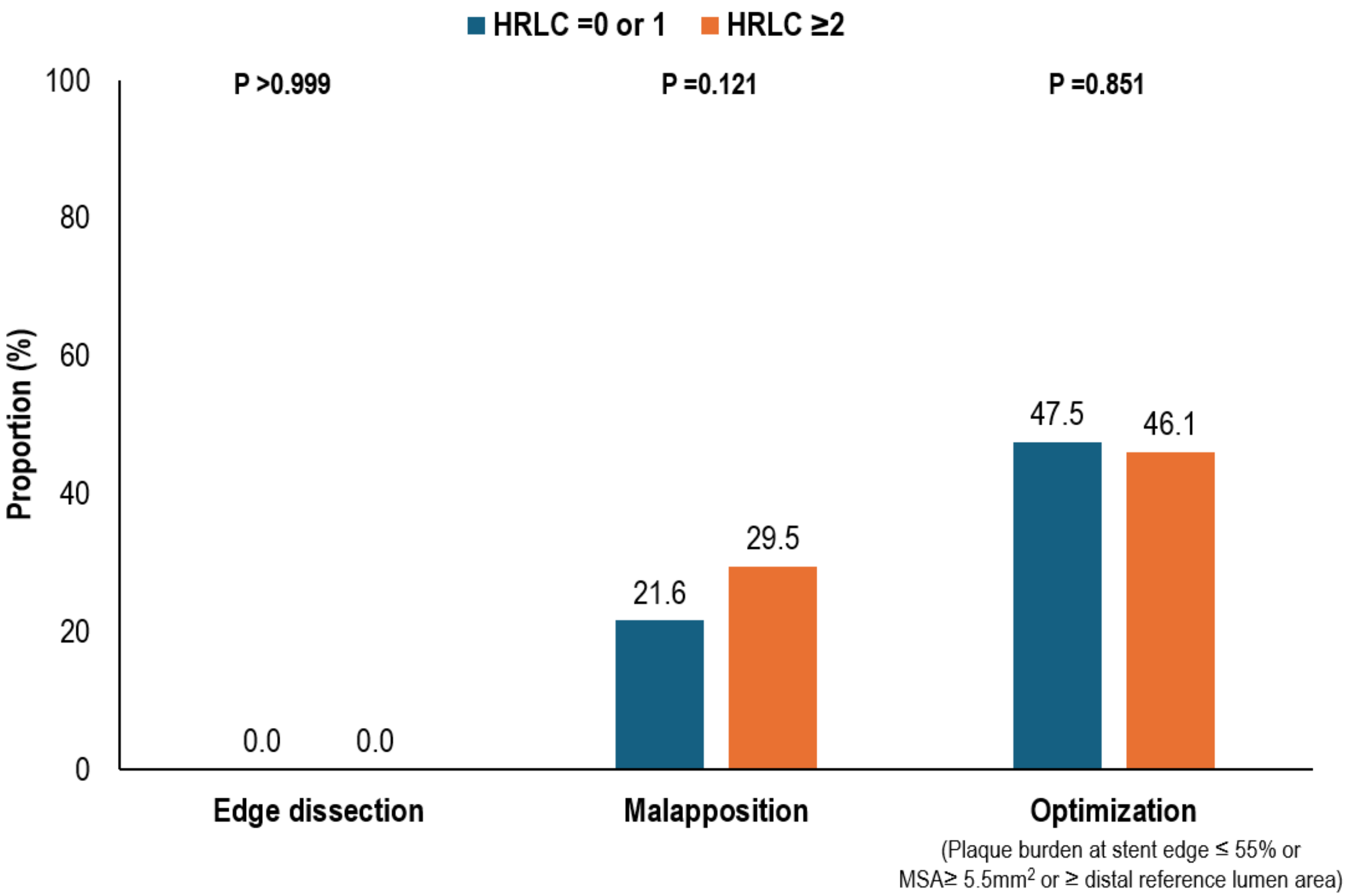

HRLC were % diameter stenosis >70%, diffuse long disease (lesion length ≥38 mm), ostial lesion, heavy calcification, and true bifurcation. Abbreviations: HRLC, high-risk lesion characteristics; IVUS, intravascular ultrasound.

Figure S6. Distribution of lesion characteristics according to ≥2 HRLC among vessels with TVF in the AngioFFR group

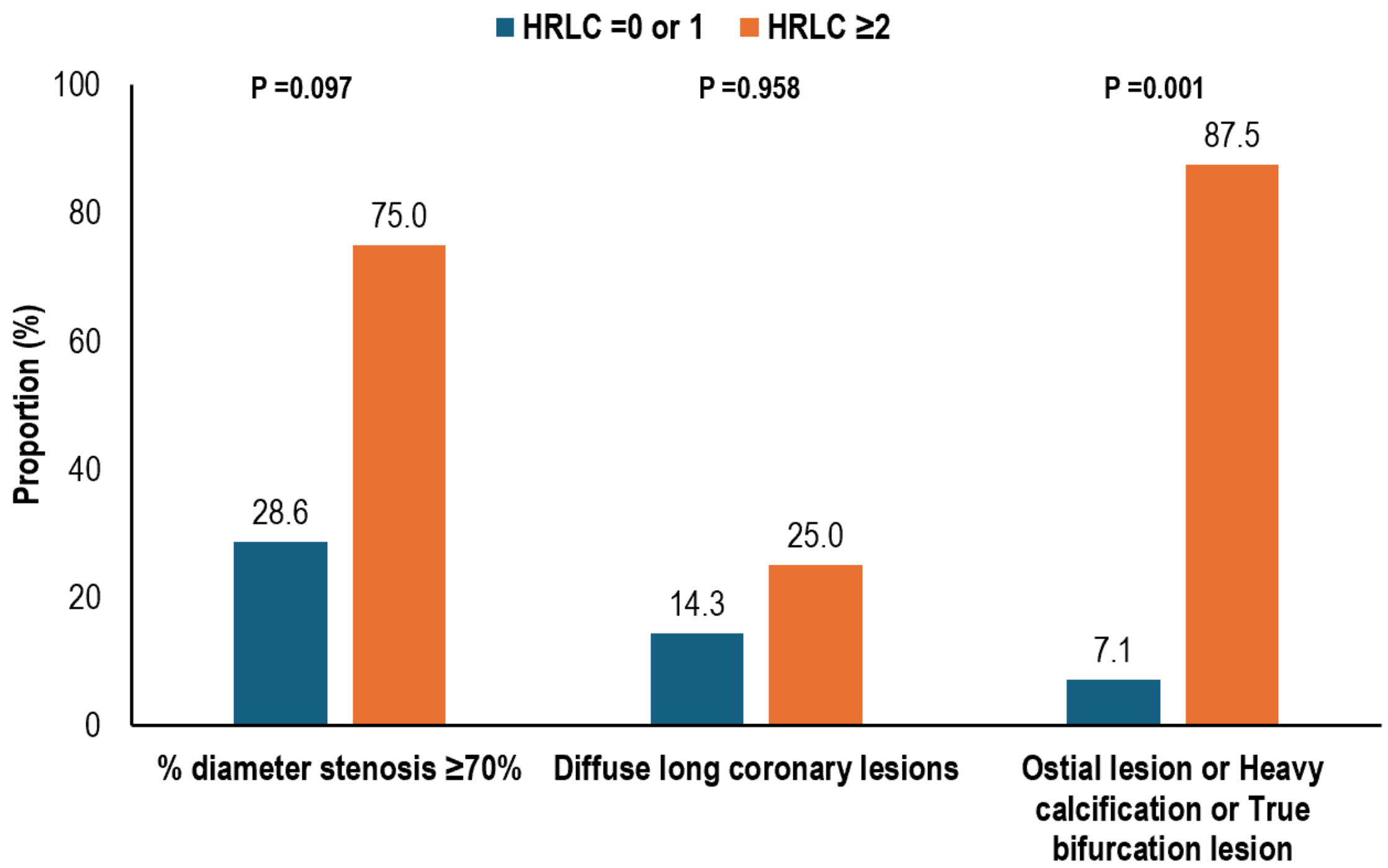

HRLC were % diameter stenosis >70%, diffuse long disease (lesion length ≥38 mm), ostial lesion, heavy calcification, and true bifurcation.
Abbreviations: AngioFFR, angiography-derived fractional flow reserve; HRLC, high-risk lesion characteristics.

3. Supplemental Tables

Table S1. Association of high-risk lesion characteristics with clinical outcomes

Number of high-risk lesion characteristics (HRLC)	Cumulative event, n (%)	Unadjusted HR (95% CI)	P-value	Adjusted HR* (95% CI)	P-value
**Cardiac death**					
HRLC =0	5/991 (0.5)	Ref	NA	Ref	NA
HRLC =1	4/584 (0.7)	1.35 (0.33 – 5.63)	0.676	1.34 (0.33 – 5.38)	0.678
HRLC ≥2	2/274 (0.8)	1.47 (0.26 – 8.23)	0.663	1.64 (0.32 – 8.31)	0.678
**Target vessel myocardial infarction**					
HRLC =0	0/991 (0.0)	Ref	NA	Ref	NA
HRLC =1	1/584 (0.2)	NA	NA	NA	NA
HRLC ≥2	0/274 (0.0)	NA	NA	NA	NA
**Target vessel revascularization**					
HRLC =0	11/991 (1.2)	Ref	NA	Ref	NA
HRLC =1	8/584 (1.4)	1.23 (0.49 – 3.06)	0.657	1.22 (0.48 – 3.08)	0.681
HRLC ≥2	10/274 (3.9)	3.35 (1.43 – 7.86)	0.005	3.25 (1.36 – 7.72)	0.016

*, adjusted for left anterior descending artery and use of μQFR; P-values were corrected for multiple comparisons using the Benjamini–Hochberg method.
High-risk lesion characteristics were % diameter stenosis >70%, diffuse long disease (lesion length ≥38 mm), ostial lesion, heavy calcification, and true bifurcation.
Abbreviations: CI, confidence interval; HR, hazard ratio; HRLC; high-risk lesion characteristics; μQFR, Murray law–based quantitative flow ratio; TRS 2°P, TIMI risk score for secondary prevention.

Table S2. Comparison of lesion characteristics between AngioFFR and IVUS-favoring subgroups

	AngioFFR favoring group (Linear predictor* ≤ -0.10) (n=563)	Indeterminate stratum (-0.10 <Linear predictor* ≤ 0.07) (n=376)	IVUS favoring group (Linear predictor* > 0.07) (n=910)	P-value
% Diameter stenosis	50.1±7.6	60.7±5.5	69.9±8.1	<0.001
Lesion length, mm	22.8±14.7	23.9±14.5	23.1±13.0	0.498
Heavy calcification, n (%)	7 (1.2)	23 (6.1)	236 (25.9)	<0.001
True bifurcation, n (%)	0 (0.0)	0 (0.0)	133 (14.6)	<0.001
Ostial lesion, n (%)	0 (0.0)	0 (0.0)	58 (6.4)	<0.001

*, Linear predictor was derived from the marginal Cox model including % diameter stenosis, lesion length, ostial lesion, heavy calcification, and true bifurcation.
Abbreviations: AngioFFR, angiography-derived fractional flow reserve; CI, confidence interval; IVUS, intravascular ultrasound.
